# Linking exogenous foliar application of glycine betaine and stomatal characteristics with salinity stress tolerance in cotton (*Gossypium hirsutum* L.) seedlings

**DOI:** 10.1186/s12870-021-02892-z

**Published:** 2021-03-20

**Authors:** Abdoul Kader Mounkaila Hamani, Shuang Li, Jinsai Chen, Abubakar Sunusi Amin, Guangshuai Wang, Shen Xiaojun, Muhammad Zain, Yang Gao

**Affiliations:** 1 Farmland Irrigation Research Institute, Chinese Academy of Agriculture Sciences/Key Laboratory of Crop Water Use and Regulation, Ministry of Agriculture and Rural Affairs, Xinxiang, Henan 453002 People’s Republic of China; 2 Graduate School of Chinese Academy of Agricultural Sciences, Beijing, 100081 People’s Republic of China

**Keywords:** Chlorophyll fluorescence, Cotton, Glycine betaine, Photosynthesis; salinity, Stomata

## Abstract

**Background:**

Glycine betaine (GB) plays a crucial role in plants responding to abiotic stresses. Studying the physiological response of cotton seedlings to exogenous GB under salt stress provides a reference for the application of GB to improve the resistance of cotton seedlings under salt stress. The purpose of this research is to examine the impacts of foliar-applied GB on leaf stomatal structure and characteristics, gas exchange and chlorophyll fluorescence characteristics and plant growth indicators of *Gossypium hirsutum* L. under NaCl stress conditions.

**Results:**

Under the salinity of 150 mM, the four concentrations of GB are 0, 2.5, 5, and 7.5 mM, and the control (CK) was GB-untreated non-saline. Salt stress negatively affected leaf stomata as well as gas exchange and chlorophyll fluorescence and decreased plant growth parameters of cotton seedlings. The treatment with 5 mM GB significantly increased the evolution of photosynthetic rate (*P*_*n*_), transpiration rate (*T*_*r*_), intracellular CO_2_ concentration (*C*_*i*_) and stomatal conductance (*g*_*s*_) compared to the GB-untreated saline treatment. The Exogenous foliar-applied GB has sustainably decreased the carboxylation efficiency (*P*_*n*_/*C*_*i*_) and water use efficiency (WUE). The concentration of 5 mM GB leads to a significant improvement of leaf stomatal characteristics. The leaf gas exchange attributes correlated positively with stomatal density (SD), stomatal length (SL) and stomatal with (SW).

**Conclusion:**

The overall results suggested that exogenous foliar supplementation with GB can effectively alleviate the damage of salt stress to cotton seedlings. The effect of applying 5 mM GB could be an optional choice for protecting cotton seedlings from NaCl stress through promoting the stomatal functions, photosynthetic activities and growth characteristics.

## Background

Plants often faced several environmental stresses that badly results in decreased plant growth and productivity [1]. Salinity has become a severe problem of agriculture, which is restricting crop productivity worldwide. It is estimated that about 50% of land loss will be observed globally due to the destructive effects of soil salinization by Wang et al. [2]. Generally, when soluble salts increased, they induced three kinds of threats to plants by creating hurdles for roots to take water in, like drought (osmotic effect), ion toxicity, and antagonistic effects of saline solutions. Salinity disturbs the energy and lipid metabolism, photosynthesis, and protein synthesis leading to stunted growth, wilting, or ultimately, death [3, 4]. Successful adaptation to salinity stress relies on many physiological variations [5]. Usually, plants build up some mechanisms to endure salts’ existence within cells or to exclude if from their cells. How crops respond to salt stress and how productive their strategies are varied among the different species [6].

Many kinds of osmolytes, including amino acids, soluble sugars, proline, and GB are produced by salt-resistant plants in an excess amount to adjust the salinity [7]. This osmotic regulation sustains sub-cellular structures and diminishes the oxidative injures because of ROS-induced elevated salinity stress [8]. Endogenously biosynthesis of GB is known as a response to stresses. The extent of biosynthesized GB mostly relies on the degree of salinity resistance [9]. The plants that are not capable of GB accumulation can resist salinity by exogenous foliar supplementation with GB [10, 11]. Further studies are required to clear the current controversy about GB function in resistance to salinity stress. Possibly, the role of the said osmolyte is based on the studied species and varieties.

One interesting feature of GB-mediated plant resilience to environmental stresses is the competence of GB produced in chloroplast to defend the photosynthetic apparatus by protecting the lipids and enzymes that are essential to sustain the optimum and linear electron flow via thylakoid membranes and sustain CO_2_ absorbance as well [12, 13]. Furthermore, an important and defensive function of GB inside the chloroplasts is photosystem II (*PSII*) stability, which remained the most susceptible compound of photosynthetic apparatus, also suggested to have a vital function in plants photosynthetic response to different environmental constraints [14]. Many researchers have reported stabilizing and protecting foliar-applied GB on the non-activation of *PSII* complex due to stress [10]. Previous investigations to correlate stomatal conductance (*g*s) and salt tolerance in barley have found positive relationships between yield and *g*s under salinity stress conditions [15]; Similar observations were made in various plant species [16]. However, changes in stomatal pore aperture or altered stomatal density (SD) could result in variations in *g*s. E Brugnoli and M Lauteri [16] reported that under high saline condition and changes in SD, plants can use a fundamental mechanism to optimse water use efficiency, as reducing the SD would be beneficial to plants subjected to osmotically stress. However, to the higher of our knowledge no study has linked NaCl stress-induced changes in SD with salinity tolerance in cotton seedlings using exogenous foliar supplementation of GB.

Cotton is one of *Gossypium* species considered to be of a global agricultural importance [17]. In northwest China and Central Asia, cotton production is limited by salinity stress caused by secondary soil salinization. However, still scarce information is available on exogenous supplementation with GB to cotton plants under saline conditions. Therefore, concidering the importance of compatible osmolyte and its action as an osmoprotectant in plants under salinity stress. This study is designed to figure out the effects of exogenous foliar-applied GB at different concentrations on the stomatal response of one cotton genotype irrigated with saline water. The current study could contribute to better understand the roles of exogenous sprayed GB on cotton seedlings stomatal functions under salinity stress.

## Results

### Effects of foliar-applied GB on gas exchange evolution and chlorophyll fluorescence

**Fig. 1:**
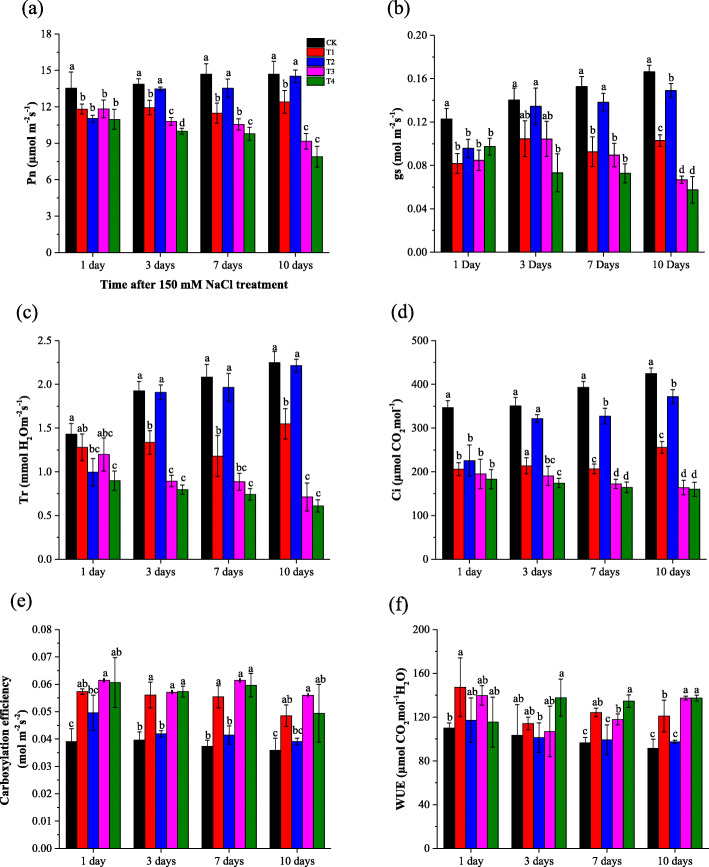
Effect of foliar-applied GB on salt-stressed cotton seedlings *P*_*n*_*, g*_*s*_*, T*_*r*_*, C*_*i*_*, Carboxylation efficiency (P*_*n*_*/C*_*i*_*) and WUE (P*_*n*_*/g*_*s*_*)* are a, b, c, d, e and f respectively. CK = control; T1 = 2.5 mM GB; T2 = 5 mM GB; T3 = 7.5 mM GB and T4 = 150 mM NaCl. Data are Mean ± standard deviation (*n* = 3). Different alphabets on top of error bars represent significant differences (*p* < 0.05)

After 10 days of 150 mM NaCl treatment, salinity significantly reduced the cotton leaf photosynthesis parameters, including *P*_*n*_*, C*_*i*_*, g*_*s*_*,* and *T*_*r*_, which significantly decreased (Fig. 1a, b, c, d), compared to CK*. P*_*n*_*, T*_*r*_*,* and *g*_*s*_ in the GB-treated saline treatments, mainly (5 mM GB) were significantly higher than in GB-untreated saline treatment after initially being applied GB for 10 days. Instantaneous carboxylation efficiency (*P*_*n*_*/C*_*i*_) and water use efficiency (WUE) were sustainably decreased during the 10 days of 150 mM saline water treatment when compared the GB-untreated non-saline to the GB-untreated saline treatments (Fig. 1e, f). From the 3rd day to 10th day of exogenous foliar treatment with GB under saline condition, the concentration of 5 mM GB was more effective in decreasing instantaneous *P*_*n*_*/C*_*i*_ and WUE.

The chlorophyll fluorescence parameters showed significant differences among the different treatments due to the foliar application of three different levels of GB under salt stress (Table 1). *F*_*v*_/*F*_*m*_, *Φ*_*PSII*_ [18], and *∆F*/ ${F}_m^{\prime }$ had a similar trend, which decreased sustainably by 6.56, 5.07 and 32.1%, respectively, under 150 mM salinity alone compared to CK, all of them significantly increased with the foliar supplementation with 5 mM GB (Table 1). Only the value of *F*_*v*_/*F*_*m*_ in T4 (0.786) was less than 0.80 with a significant difference with CK, T1 and T2, suspecting that photo-inhibition might have occurred in T4 [18]. More significant differences among treatments were observed for *∆F*/ ${F}_m^{\prime }$ than for *F*_*v*_/*F*_*m*_.

**Table 1:** The chlorophyll fluorescence parameters in the different treatments

Treatment	*∆F/* ${F}_m^{\prime }$	*Φ*_*PSII*_	*Fv/Fm*	*qN*	*qP*	*NPQ*
CK	0.747 ± 0.006 a	0.352 ± 0.015 a	0.828 ± 0.003 a	0.765 ± 0.019 ab	0.459 ± 0.058 b	1.726 ± 0.128 bc
T1	0.715 ± 0.027 ab	0.271 ± 0.015bc	0.825 ± 0.007 a	0.739 ± 0.007 b	0.496 ± 0.019 b	1.554 ± 0.079 c
T2	0.730 ± 0.005 a	0.310 ± 0.015ab	0.829 ± 0.004 a	0.732 ± 0.017 b	0.490 ± 0.026 b	1.538 ± 0.087 c
T3	0.707 ± 0.012 b	0.257 ± 0.023 c	0.801 ± 0.015 ab	0.777 ± 0.017 ab	0.536 ± 0.046ab	1.748 ± 0.096 bc
T4	0.698 ± 0.005 b	0.239 ± 0.027 c	0.786 ± 0.017 b	0.824 ± 0.042 a	0.559 ± 0.033 a	2.260 ± 0.419 a

*Note*: *∆F/* ${F}_m^{\prime }=\mathrm{Effective}\ \mathrm{quantum}\ \mathrm{efficiency}\ \mathrm{of}\ \mathrm{photosystem}\ \mathrm{II},$ *Φ*_*PSII*_ = Actual photochemical efficiency of photosystem II, *Fv/Fm* = Maximal photochemical efficiency of photosystem II, *qN* = non-photochemical quenching, *qP* = photochemical quenching of variable chlorophyll, *NPQ* = non-photochemical quenching Data are Mean ± standard deviation (*n* = 3). CK = control; T1 = 2.5 mM GB; T2 = 5 mM GB; T3 = 7.5 mM GB and T4 = 150 mM NaCl. Different alphabets in each sub-column represent significant differences (*p* < 0.05)

The *qP, qN*, and *NPQ* showed a contrary trend with *F*_*v*_/*F*_*m*_, *∆F*/ ${F}_m^{\prime }$ and *Φ*_*PSII*_, they were sustainably higher in the GB-untreated saline treatment (T4) compared to the well-watered control, but all of them significantly decreased in T1 and T2 due to the foliar application of GB (Table 1). The differences in *qN* and *qP* between the GB-treated saline treatments were insignificant (Table 1). The differences in *NPQ* between control and GB-treated saline treatments were insignificant, but all the treatments, when compared to the saline treatment alone, showed significant differences. The *NPQ* showed a maximum value of 2.260 in T4.

### Effects of foliar-applied GB on stomatal structure and characteristics

**Fig. 2:**
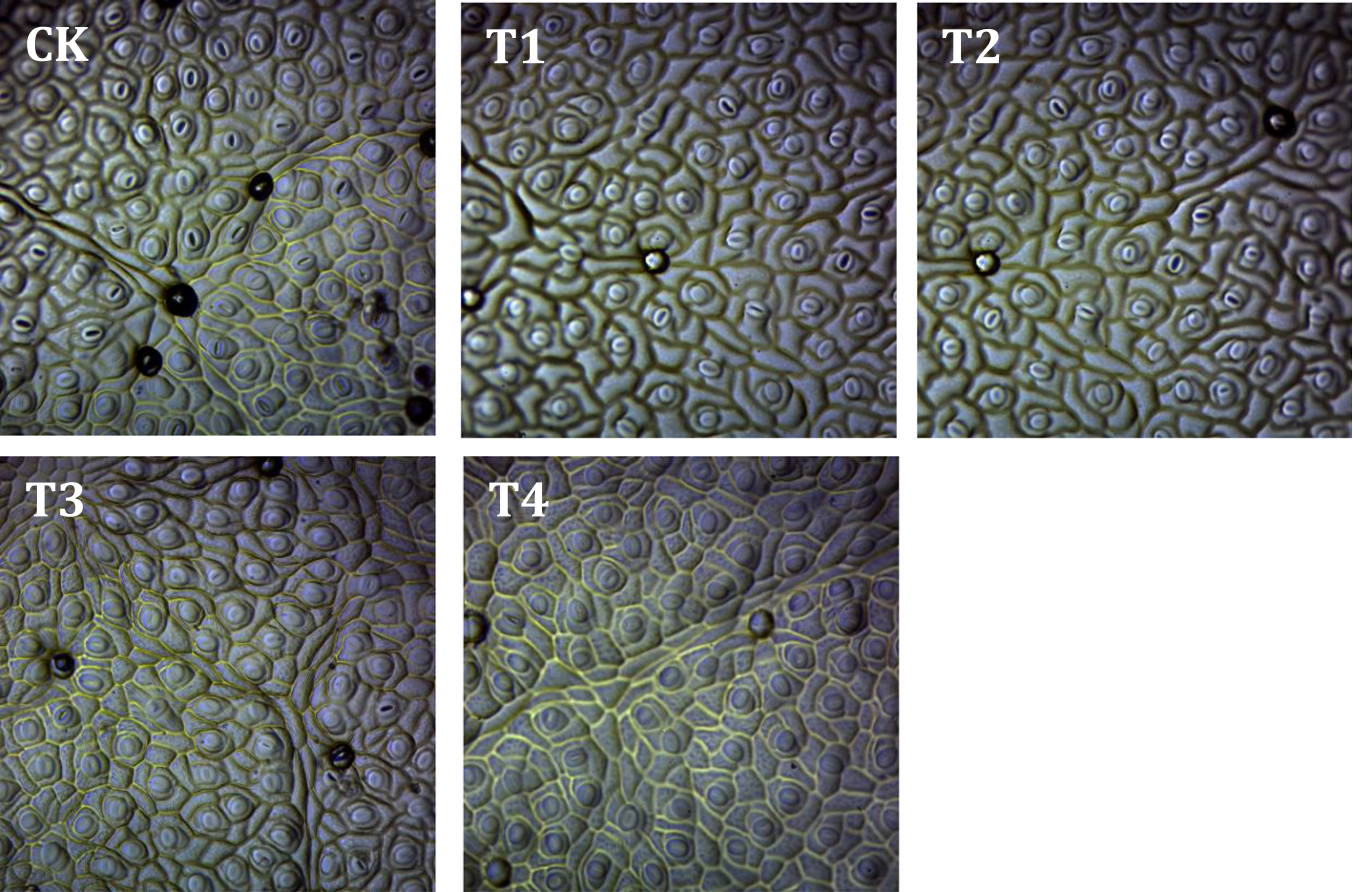
Effect of foliar-applied GB on stomatal morphology of salt-stressed cotton seedlings. CK = control; T1 = 2.5 mM GB; T2 = 5 mM GB; T3 = 7.5 mM GB and T4 = 150 mM NaCl

**Fig. 3:**
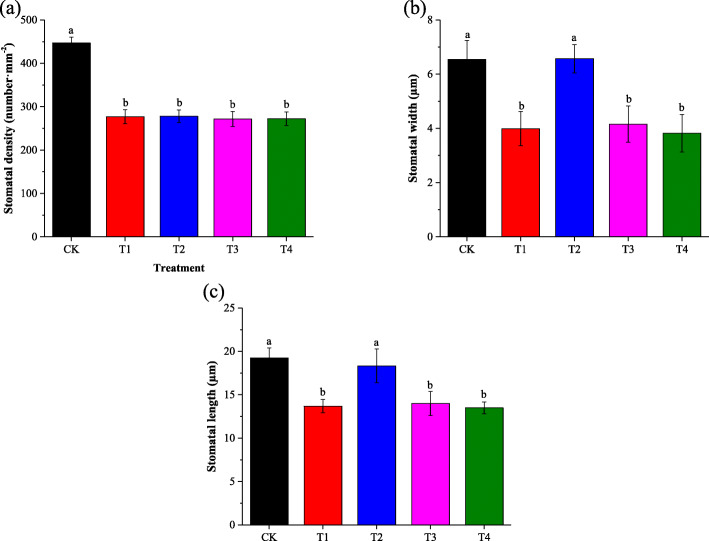
Effect of foliar-applied GB on salt-stressed cotton seedlings stomatal density, stomatal width, and stomatal length are a, b, and c respectively. CK = control; T1 = 2.5 mM GB; T2 = 5 mM GB; T3 = 7.5 mM GB and T4 = 150 mM NaCl. Data are Mean ± standard deviation (*n* = 3). Different alphabets on top of error bars represent significant differences (*p* < 0.05)

Figure 2 showed the effect of different exogenously applied GB concentrations on seedlings stomatal structure under 150 mM salt stress. Under the condition of NaCl alone without exogenous treatment (T4), the small pores on the surface of leaves (stomatal) are more closed compared to that on the control treatments’ leaves. The foliar supplementation with 2.5 and 5 mM GB increased the opening diameter of stomata under salt stress when compared to the non-exogenously treated saline (Fig. 2). Salinity has significantly decreased stomatal characteristics including, stomatal density, length and width when compared CK to the exogenously untreated saline treatment (Fig. 3). Among all the foliar-applied GB levels, only the concentration of 5 mM GB has significantly increased the stomatal length and width under salt stress condition (Fig. 3b, c). But, none of the exogenous GB levels had a significant positive effect on leaf stomatal density (Fig. 3a).

### Effects of foliar-applied GB on the evolution of growth parameters

**Fig. 4:**
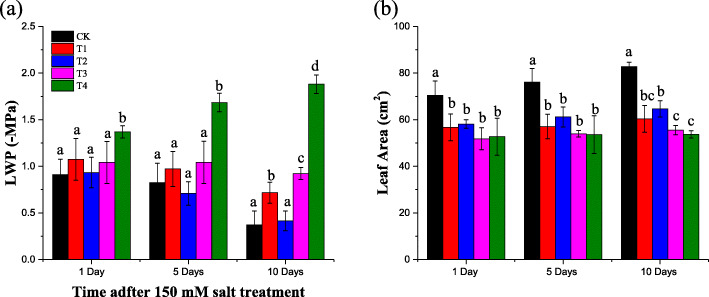
Effect of foliar-applied GB on salt-stressed cotton seedlings LWP and leaf area are a and b, respectively. CK = control; T1 = 2.5 mM GB; T2 = 5 mM GB; T3 = 7.5 mM GB and T4 = 150 mM NaCl. Data are Mean ± standard deviation (*n* = 3). Different alphabets on top of error bars represent significant differences (*p* < 0.05)

During the 10 days of salt treatment, plant growth characteristics such as LWP and leaf area were significantly decreased comparing the well-watered treatment with the GB-treated saline treatments (Fig. 4). Up to 10 days of exogenous foliar supplementation with GB, the value of LWP remained significantly lower in the GB-treated saline treatments (Fig. 4a). Only the treatment with 5 Mm GB had a significant positive effect on leaf area at the 10th day of treatment GB foliar treatment under salt stress (Fig. 4b).

### Effects of foliar-applied GB on GB, proline, soluble sugar, and protein content

**Fig. 5:**
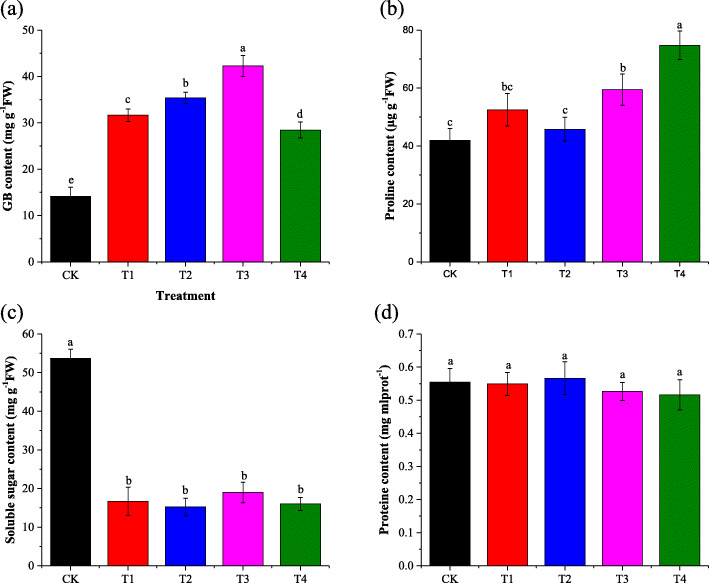
Effect of foliar-applied GB on salt-stressed cotton seedlings GB content, proline content, soluble sugar content, and protein content are a, b, c and d respectively. CK = control; T1 = 2.5 mM GB; T2 = 5 mM GB; T3 = 7.5 mM GB and T4 = 150 mM NaCl. Data are Mean ± standard deviation (*n* = 3). Different alphabets on top of error bars represent significant differences (*p* < 0.05)

GB, proline, soluble sugar and protein content measured in cotton leaves after harvesting are presented in Fig. 5. The endogenous concentrations of GB [18] and proline were significantly increased as response to salt stress. While the soluble suger content was dramaticaly decreased under saline condition when compared the salt-stressed alone with control. Exogenous foliar suplementation with GB has significantly increased the endogenous GB content (Fig. 5a) and decreased proline content (Fig. 5b) when compared the exogenous foliar treatments with the salt-stressed alone. The soluble suger concentration was insignificantly affected by the exogenously-applied GB (Fig. 5c). Treatment with 150 mM salt without exogenous and treatment with exogenous GB under 150 mM salt stress had an insignificant effect on protein content(Fig. 5d). Differences in protein content in cotton leaves of all treatments were insignificant.

### Relationship of endogenous GB content and gas exchange parameters with stomatal characteristics

**Fig. 6:**
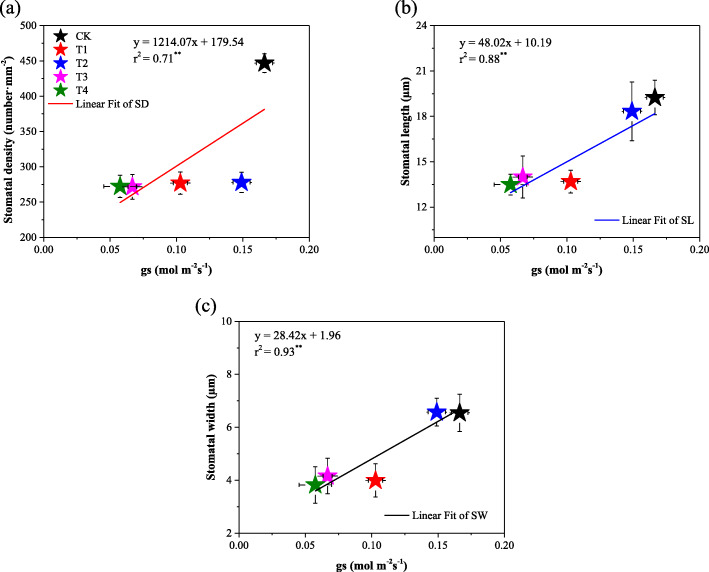
Relationship between stomatal characteristics (a) stomatal density (SD), (b) stomatal length (SL) and (c) stomatal width (SW) and stomatal conductance (*gs*). CK = control; T1 = 2.5 mM GB; T2 = 5 mM GB; T3 = 7.5 mM GB and T4 = 150 mM NaCl. Data are Mean ± standard deviation (*n* = 3), * and ** indicate significance levels of *P* < 0.05 and *P* < 0.01, respectively

**Fig. 7:**
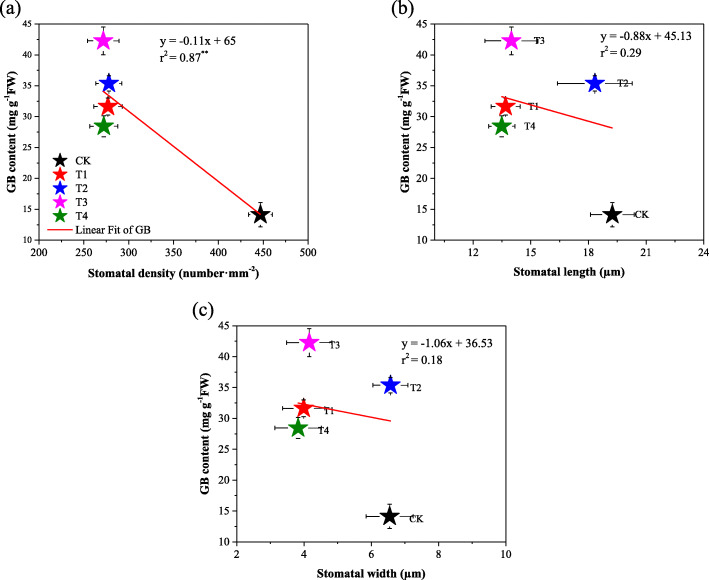
Relationship between endogenous GB content and (a) stomatal density (SD), (b) stomatal length (SL) and (c) stomatal width (SW). CK = control; T1 = 2.5 mM GB; T2 = 5 mM GB; T3 = 7.5 mM GB and T4 = 150 mM NaCl. Data are Mean ± standard deviation (*n* = 3), * and ** indicate significance levels of *P* < 0.05 and *P* < 0.01, respectively

Cotton seedlings leaf gas exchange parameters were negatively affected by salinity, but GB leads a significant improvement in cotton leaf gas exchange parameters when applied to leaves. High significant positive Persons’ correlation was observed between leaf gas exchange parameters. Cotton seedlings leaf stomatal characteristics showed high significant positive correlated with gas exchange parameters (Table 2). All stomatal characteristis (SD, SL, and SW) showed siginificant positive relashioship with *gs* (Fig. 6). On another hand, the leaf endogenous concentration of GB significantly negativelly correlated with leaf stomatal density (SD), while there was no significant relashionship between GB content and stomatal length (SL) and stomatal wigth (SW) (Fig. 7).

**Table 2:** Correlation matrix between gas exchange parameters and Stomatal characteristics

	*P*_*n*_	*g*_*s*_	*C*_*i*_	*T*_*r*_	SL	SW	SD
*P*_*n*_		96***	92***	99***	59**	71**	68**
*g*_*s*_			99***	95***	72**	85**	73**
*C*_*i*_				95***	72**	87**	72**
*T*_*r*_					62**	79**	70**
SL						65**	56**
SW							89***

*Note*. *P*_*n*_ = Net photosynthetic rate, *g*_*s*_ = stomatal conductance, *C*_*i*_ = intracellular CO_2_ concentration, *T*_*r*_ = Transpiration rate, *SD* = Stomatal density, *SL* = Stomatal length, *SW* = stomatal width. *, **, and *** indicate significance levels of *P* < 0.05, *P* < 0.01, and *P* < 0.001 respectively

## Discussion

Exogenous foliar supplementation with compatible solutes is commonly known to trigger tolerance mechanism of plants against various abiotic stresses conditions. Exogenous foliar-applied GB can easily penetrate through leaves and be transferred to other parts of plants, where it would contribute to ameliorate plants resistance to stress conditions [19]. In the current study, we have investigated cotton seedlings’ physiological response to short-time exogenous foliar treatment with GB under 150 mM saline condition. In this connection, we mostly focused on the effect of foliar-applied GB on seedlings’ stomatal activity attributes under saline conditions. Studies involving plant response to saline conditions have been conducted using physiological, biochemical, molecular, and proteomic methods [20, 21]. The alteration of some key physio-biochemical processes such as tissue water potential, photosynthetic, and chlorophylls efficiencies resulting from salt stress when harmonized with excessive production and the reactions of ROS leads to an increase in oxidative stress [22].

Plants’ stomatal are the source of exchange of air and water between plant leaves and the atmosphere. Stomatal are also one of the main factors affecting the photosynthetic and transpiration process of plants. Plants can adjust stomatal distribution according to changes in environmental factors. Stomatal density and stomata size, make plants lose less water condition, get the most CO_2_ down [23]. Many factors involved in the relationship between plant water status and stomatal functionality [24]. Salt stress condition in this study has decreased the stomatal opening and density of cotton leaves. Similarly, a research reported that under saline conditions, plants have to close their stomata due to water loss [25].

Leaf gas exchange regulation is known to be an important aspect of improving plant resistance to various environmental stresses, including salt stress [18]. Salt stress is reputed to photosynthesis inhibition in many plant species as mentioned in researches that *T*_*r,*_ *P*_*n*_, and *g*_*s*_ decrease under saline conditions [26, 27]. Regulating leaf gas exchange capacity is vital for plants’ resistance to various environmental and non-environmental stress conditions [28]. Reports showed that plants implement that strategy to regulate several photosynthetic attribute, including *C*_*i*_*, T*_*r*_*, P*_*n*_*,* and *g*_*s*_ [29, 30]. We hypothesized that the positive effect of GB on *g*_*s*_ could simply be mechanical, as observed that GB increase the proportion of bound water in the cell structures as well as the turgor pressure in the guard cells of stomatal [31]. Moreover, we observed that foliar-applied GB increased *g*_*s*_ and allows more efficient gas exchange. Exogenous foliar supplementation with GB was known to enhance growth, *P*_*n*_, leaf water content, and *PSII* of salt-stressed maize plants [32]. In our study, we observed that *P*_*n*_, *C*_*i*_, *T*_*r*_, and *g*_*s*_ sustainably decreased under 150 mM salinity stress (Fig. 1), but the foliar supplementation with GB statistically enhanced them under salt stress. As observed, the WUE and the carboxylation efficiency were sustainably increased as response NaCl treatment. The increase of WUE under150 mM salt condition could explain that cotton seedlings might utilize the water efficiently, in order to sustain the growth. In harmony with our findings, it was observed an increase in the WUE in sugar beet under a moderate saline condition [33]. Furthermore, exogenous foliar application of GB improved growth, *Pn*, and WUE in maize plants subjected to salt stress [32].

Leaf stomatal characteristics and gas exchange parameters negatively responded to the short-time stress of 150 mM NaCl. The decrease of leaf gas exchange attributes might be attributed to stomatal restriction, including stomatal closure and stomatal density (SD) reduction. A recent study reported that, salt stress inhibits the photosynthetic process of plant leaves mainly including stomatal restriction [23]. As expected our study results showed a significant positive relationship between gas exchange attributes and stomatal characteristics (Table 2). Persons’ correlation revealed a significant positive relationship between cotton leaf SD and leaf stomatal conductivity, reflecting that the variation of stomatal density directly affects the stomatal conductivity. In this study, we found the exogenous foliar application of GB to be a suitable way to improve cotton leafs stomatal opening (Fig. 2) which finally turns to improve gas exchange attributes (Fig. 2) under saline conditions. The current study showed that cotton leaf stomatal characteristics (SD, SL and SW) have significant positive relationships with *gs* (Fig. 6). Furthermore the cotton leaf endogenous GB content correlated negatively with SD (Fig. 7), meaning that SD sustainably decreased with the NaCl-induced GB accumulation.

Chlorophyll fluorescence is a key signal reflecting the plant growth inhibition by a saline environment. A suitable and rapid method for detecting and measuring plant resistance to abiotic stresses, is evaluating the integrity of leaf photosynthesis based on chlorophyll fluorescence [18]. When higher plants are subjected to salinity stress, *F*_*v*_/*F*_*m*_ of non-salt tolerant plants is reduced [34], but that of salt-resistant plants is enhanced [35]. In our study, saline conditions significantly decreased PSII activity [18] as also reported in radish [36], tomato [37], sunflower [38], and wheat [39]. But upon the application of GB, PSII activity was increased for the saline conditions [18]. Similar results were observed for eggplant [40], but opposite to the result obtained in watermelon [41]. Therefore, the reduction in *F*_*v*_/*F*_*m*_ can be considered as a symbol of photo-inhibition when plants are subjected to salinity. It is usually noticed that photo-inhibition results in a vast reduction in the actual quantum yield (*ΔF*/ ${F}_m^{\prime }$), which response to salinity stress is similar to that of *F*_*v*_/*F*_*m*_ but more significantly. It has been reported that GB application do not affect *PSII* photochemistry (*F*_*v*_/*F*_*m*_) maximum efficiency [19]. Enhancements in *gs* and *PSII* is associated with the improvement in *Pn* of salt-stressed maize plants treated with GB. The *F*_*v*_/*F*_*m*_ remained approximately constant (0.80 ∼ 0.85) for various plant species growing in normal conditions [42]. In this study, only the *F*_*v*_/*F*_*m*_ of T4 (0.786 ± 0.017) was below 0.80 [18], meaning that it was inhibited more significantly by salinity than the other four treatments (Table 1). We concluded that the foliar application of GB significantly increased the *F*_*v*_/*F*_*m*_ of cotton under salt stress conditions.

Moreover, the decrease of *F*_*v*_/*F*_*m*_ is due to the improvement of *Pn* or heat dissipation, while the photosynthetic activity of plants can be computed by photochemical quenching (*qP*) and the capacity of plants to dissipate excess light energy can be reflected by non-photochemical quenching (*qN* or *NPQ*). In our study, the *qP*, *qN*, and *NPQ* were higher in the GB-untreated saline treatment (Table 1). The foliar application of GB significantly decreased the values of *qP* and *qN* in T1 and T2 and that of *NPQ* in T1, T2, and T3, but for *qN* the differences were insignificant between the GB-treated saline treatments and the GB-untreated saline treatment (Table 1). A study on maize showed a significant increase in *NPQ* under saline conditions. But, less increase was observed in *NPQ* in salt-stressed maize plants as response to exogenous foliar application of GB [32].

Accumulations of GB, proline, protein and soluble sugar have been determined in various plant species subjected to salinity stress. Salinity stress has most of the time increased the endogenous concentrations of GB in shoot of many plant, while, endogenous GB content insignificantly vary in root of many plants [43]. It was observed an increase of proline content in the rice salt-tolerant genotype with exogenous foliar application of GB under salt stress conditions [44]. On the contrary, foliar-applied GB reduced the proline content in S-24 salt-tolerant wheat and IR28 salt-sensitive rice and MH-97 salt-sensitive genotypes under saline conditions [44, 45]. Similarly proline content was decreased in spinach and rapeseed as response to exogenously applied GB under saline conditions [46]. In our study, it was observed that 150 mM NaCl condition caused an increase of endogenous GB [18] and proline content and a decrease of soluble sugar content (Fig. 5). We hypothesized that the cotton genotype used in this experiment is able itself to synthesize a considerable amount of GB under saline conditions. However, exogenous foliar application of GB resulted in further increase in GB and soluble sugar content but decrease in proline concentration. In a harmony with our findings, earlier studies found exogenous foliar-applied GB to enhance soluble sugar content under saline conditions [47–49]. In contradiction with our results, exogenous foliar application of GB caused an increase in rice under saline conditions [44]. It has been reported that various plants species accumulate greater concentrations of protein under saline conditions [43]. In the current study, the accumulation of protein in cotton leaves was insignificantly affect by salinity.

## Conclusion

GB has been widely used in agriculture as a compatible solute, which can usually improve abiotic stress tolerance in crops. Our results concluded that foliar supplementation with GB could be a suitable for improving the leaf stomatal structure and characteristics of cotton seedlings. This study demonstrated that under NaCl stress condition, the increase of stomatal characteristics by foliar supplementation with GB leads to a rapid improvement of gas exchange and chlorophyll fluorescence in *Gossypium hirsutum* L. seedlings. In summary, the improved salt tolerance by exogenous application of GB could be due to its contribution to significantly improve gas exchange parameters. The strategic and economic level of exogenous GB should be fixed for alleviating the adverse effect of NaCl stress on cotton. This study recommends foliar spraying of 5 mM GB for mitigating the NaCl-induced damages in cotton seedlings by improving leaf stomatal responses to saline conditions. Thus, further studies should examine the biochemical and molecular mechanisms of foliar-applied GD and endogenously accumulated GB in order to find out the similarities and differences between endogenously synthesised and foliar-applied and GB.

## Methods

### Plant materials

The current research was conducted during 45 days from sowing to harvesting in a controlled environmental chamber at the Research site of Farmland Irrigation Research Institute, Graduate School of Chinese Academy of Agricultural Sciences (FIRI-GSCAAS), located in Qiliying, Xinxiang city, Henan Province, North China. The chamber conditions were as follows: the temperature for day/night of 30/20 °C, photoperiod of 14 h [06:00–20:00 h Beijing Standard Time (BST)], the density photosynthetic photon flux was 350 μmol m^− 2^ s^− 1^ and relative humidity in the range of 50–60%. Uniform seeds of *Gossypium hirsutum* L., cultivar Xinluzhong-37, were obtained and disinfected in 0.3% hydrogen peroxide for 30 min and finally washed thrice with deionized water. To insure the germination, cotton seeds were sown in flat trays containing sterile sand, and one-week-old seedlings with uniform size were transplanted into plastic pots (16 cm diameter, 18 cm height, and 1 plant pot^− 1^). Each pot was filled with 2.5 kg of sterilized sand in order to avoid any nutrient effect. The transplanted seedlings were irrigated with half-strength Hoagland solution on a regular basis for 20 days after transplantation to provide seedlings the required nutrients for plant growth. On the 20th day of their growth, plants were watered with NaCl contained in Hoagland solution at 50 mM, then watered with NaCl contained in Hoagland solution at 100 mM on the 22nd day, and the desired salinity level of 150 mM was applied on the 25th day. The salt concertation of 150 mM was maintained until 10 days, and exogenous glycine betaine dissolved in deionized water was daily sprayed on the upper sides of all leaves at 5 ml per plant during those 10 days. A total number of 30 plants was used during the experiment, with 6 plants per treatment. The treatments (Table 3) were laid in a completely randomized design. Photosynthetic, plant growth, and development and chlorophyll fluorescence parameters were periodically measured on the tenth day of double treatments (exogenous GB and salinity). Plants were harvested 10 days after initially being treated with exogenous GB, to measure other physiological and biochemical characteristics as described below.

**Table 3:** The treatments arrangement

Treatment	NaCl concentrations (mM)	GB concentrations (mM)
CK	0	0
T1	150	2.5
T2	150	5
T3	150	7.5
T4	150	0

*Note*: CK = control; T1 = 2.5 mM GB; T2 = 5 mM GB; T3 = 7.5 mM GB and T4 = 150 mM NaCl

### Gas exchange and chlorophyll fluorescence

The leaf gas exchange parameters including photosynthetic rate (*P*_*n*_), intracellular carbon dioxide concentration (*C*_*i*_), transpiration rate (*T*_*r*_) and stomatal conductance (*g*_*s*_) were measured every 3 days from 09:00 am to 11:00 am BST in all treatments using the tird fully expanded leaves, during the period of exogenous GB application with the Li-6400XT portable photosynthesis system (Li-COR Inc., Lincoln, NE, USA). A single leaf was used per replication for gas exchang measurements. During measurements, reference CO_2_ concentration was equilibrated to 400 μmol mol^− 1^ with a CO_2_ mixture, and the light adjusted at a PAR of 1200 μmol m^− 2^ s^− 2^. The block temperature was fixed at 25 °C, the leaf-to-air VPD was equilibrated between 1.5 and 2.0 kPa, and the flow was fixed at 300 μmol s^− 1^.

Chlorophyll fluorescence was simultaneously measured the same days as measuring leaf gas exchange parameters using the MINI-PAM-II/R Photosynthesis Yield Analyzer. The leaves were adapted to darkness over-night to measure the initial and maximum fluorescence, (*F*_*o*_) and (*F*_*m*_) respectively, the variable chlorophyll (*F*_*v*_) was computed as *F*_*v*_ = *F*_*m*_ – *F*_*o*_ and the maximal photochemical efficiency of photosystem II (*PSII*) was expressed as *F*_*v*_/*F*_*m*_. ${F}_m^{\prime }$ was measured under the full light, while ${F}_o^{\prime }$ was measured after turning off the light, and *∆F* is the difference between *F*_*m*_ and *F*_*s*_. The variable chlorophyll fluorescence under the fluorescence condition $\Big({F}_v^{\prime }$) was computed as ${F}_v^{\prime }={F}_m^{\prime }-{F}_o^{\prime }$. *qP*, *qN* and *NPQ* were computed as *qP* = 1 – (${F}_m^{\prime }$ – F)/(${F}_m^{\prime }$ – ${F}_o^{\prime }$); *qN* = 1 – (${F}_m^{\prime }$ – ${F}_o^{\prime }$)/(*F*_*m*_ – *F*_*o*_) and *NPQ* = (*F*_*m*_ – ${F}_m^{\prime }$)/ ${F}_m^{\prime }$ respectively [50]. The actual photochemical efficiency of Photosystem II (*Φ*_*PSII*_) was computed as:

$$ {\varPhi}_{PSII}=\frac{F`m- Fs}{F`m}. $$

### Determination of leaf stomata structure and characteristics

FEI Scanning Electron Microscope Quanta 200F (Field Emission Instruments Co.) was used to captured Images of the abaxial and adaxial epidermal surface of cotton leaves. Pictures were taken under the 10x eyepiece of the OLYMPUS microscope for stomatal structure and 40x eyepiece for stomatal characteristics [51]. ImageJ 1.4.8 software was used for processing and analysis.

### Determination of plant growth parameters

Plant growth parameters such as the leaf area and LWP were measured three times in 5 day intervals during the 150 mM salinity stressing period (20–35 DAT). The leaf area was measured using a leaf area meter (model 3050A, Li-Cor Biosciences, Lincoln, NE, USA). The LWP was measured using WP4C, Dewpoint Potential Metter [52].

### Quantification of protein, GB, proline and soluble sugar

Protein content was measured by homogenizing 3 g of frozen leaf sample in 15 mL of ice-cold solution containing 100 mM Tris (pH 7.0), 10 mM D-isoascorbic acid, 20 g L-1 PVP-10 (polyvinylpyrrolidone), 1.5 g insoluble PVP, 0.1 mM EDTA, and 2 mL L^− 1^ Triton X-100 (Rohm & Haas Co., Philadelphia, PA) [53]. The homogenized sample was then filtrated with miracloth, and the extract centrifuged at 10000 g for 15 min at 4 °C. The supernatant was then loaded in 9.5 mm dialysis tube packed in sucrose crystals, and stored at 4 °C until the volume decreased to about 1 to 2 mL. One milliliter of the decreased extract was centrifuge-desalted through a 10 mL bed of Sephadex G50–300 at 1500 g for 3 min to reject component, which molecular weights are less than 15,000 [54]. A part of the eluent was analyzed immediately for catalase activity, then the remaining stored at − 70 °C for analysis of total protein content. GB was extracted from ½ g of frozen tissue using methanol-chloroform-water, and the isolation followed the ion-exchange chromatography procedure described earlier [55]. The instrumentation was as described by A Hanson and D Gage [56]. Proline was determined spectrophotometrically following the ninhydrin method described by LS Bates, RP Waldren and ID Teare [57] using Merck proline as a standard. The soluble sugar content was determined according to Yoon et al. [58] with the following modifications. Approximately 0.1 g of the sample was weighed, placed in a polypropylene tube containing 6 ml of extraction solution (80% ethanol), homogenized, incubated in a water bath at 65 C for 20 min and then centrifuged at 3500 rpm for 10 min, after which the supernatant was collected.

### Statistical analysis

One-way ANOVA was done with all data performed as mean (*n* = 3) followed by standard deviation. Significant means were separated using Duncan’s test at *p* < .05 in SPSS software of International Business Machine 19.0 (IBM SPSS, Inc., Chicago, IL, U.S.A.). Correlation analysis was performed to determine the associations between measured parameters.

## Data Availability

The datasets used and/or analysed during the current study are available from the corresponding author on reasonable request.

## Abbreviations

BST: Beijing standard time
C_i_: Intracellular CO_2_ concentration
F: Fl*uorescence in the steady-state*
F_m_: *Maximum fluorescence yield*
F_o_: *Initial fluorescence yield*
F_s_: *Steady-state fluorescence*
F_v_: Variable chlorophyll
${F}_v^{\prime }$: Variable chlorophyll fluorescence under the fluorescence condition
GB: Glycine betaine
g_s_: Stomatal conductance
LA: Leaf area
LWP: Leaf water potential
NPQ: Non-photochemical quenching
P_n_: Photosynthetic rate
qP: Photochemical quenching
qN: Non-photochemical quenching of variable chlorophyll
SD: Stomatal dendity
SL: Stomatal length
SW: Stomatal width
T_r_: Transpiration rate
